# Evaluation of Novel Immunohistochemical Biomarkers for the Diagnosis of Celiac Disease Demonstrates the Utility of TCRδ Immunostaining

**DOI:** 10.3390/diagnostics16111694

**Published:** 2026-05-30

**Authors:** Heeyeon Lee, Vrinda Shenoy, Priyanka Gopalkaje, Sam Parsons, Anuradha Kaistha, Elizabeth J. Soilleux

**Affiliations:** Department of Pathology, University of Cambridge, Tennis Court Road, Cambridge CB2 1QP, UK; heeyeon.lee@kcl.ac.uk (H.L.); vs534@cantab.ac.uk (V.S.); pg547@cam.ac.uk (P.G.); sam.parsons@nhs.net (S.P.); ak2140@cam.ac.uk (A.K.)

**Keywords:** celiac disease, diagnostics, immunohistochemistry, biomarker, intra-epithelial lymphocyte, BTNL8, NKp46, TdT, THEMIS, TCRδ

## Abstract

**Background/Objectives**: Celiac disease (CD) is a T-cell-mediated autoimmune condition, triggered by gluten ingestion. Duodenal biopsy is the gold-standard diagnosis for CD, which is often limited by interobserver variability between pathologists. Immunohistochemistry (IHC) is a powerful technique for detecting biomarkers with potential diagnostic significance. This study aims to investigate five candidate biomarkers, BTNL8, NKp46, TdT, THEMIS, and TCRδ, that might improve the reproducibility of the diagnosis of CD. **Methods**: Formalin-fixed paraffin-embedded material, surplus to diagnostic requirements, was obtained from 46 subjects (untreated CD: *n* = 21, CD treated with gluten-free diet: *n* = 5; controls: *n* = 20) and immunostained for BTNL8, NKp46, TdT, THEMIS and TCRδ. BTNL8 staining was scored on a 0–3 semi-quantitative scale. NKp46, TdT, THEMIS, and TCR delta-positive intra-epithelial lymphocytes (IELs) were quantified as mean counts per 100 epithelial cells (ECs). **Results**: TCRδ-positive IELs were markedly elevated in CD biopsies (median 9.4 IELs/100 ECs) compared to healthy controls (median 0.5 IELs/100 ECs; *p* < 0.001), with a threshold of >2.1 TCRδ-positive IELs per 100 ECs yielding an AUC of 0.94 and interobserver agreement of 0.82. NKp46 expression was also increased in CD (median 13.8 IELs/100 ECs) versus controls (median 9.6; *p* < 0.001), with >12.8 NKp46-positive IELs per 100 ECs achieving an AUC of 0.86 and interobserver agreement of 0.82. Immunostaining for the other biomarkers demonstrated less clear differences between CD and healthy controls. **Conclusions**: Corroborating several recent publications, TCRδ immunostaining provides high diagnostic accuracy and good interobserver agreement in the diagnosis of CD on duodenal biopsy, even for patients on a gluten-free diet.

## 1. Introduction

### 1.1. Overview of Celiac Disease

Celiac disease (CD) is a T-cell-mediated autoimmune condition triggered by gluten ingestion in genetically susceptible individuals [1]. CD is distinctive among autoimmune conditions as key triggering antigens, dietary gluten and the autoantigen, tissue transglutaminase (tTG), have long been known. Previously considered to be a rare disorder only affecting European children, CD is now recognized to be the most common autoimmune disorder worldwide, affecting individuals of all ages [2,3]. The estimated prevalence of CD is 1% of the general population, steadily rising in Western countries [4]. Although the number of CD diagnoses has increased over the last three decades, CD underdiagnosis persists globally, and is attributed to its variable clinical presentation and to insufficient awareness of its diverse clinical manifestations [5].

The clinical presentations of CD include gastrointestinal (GI) and non-specific symptoms that vary with age and between individuals [6,7]. “Classical” symptoms include malabsorptive symptoms such as diarrhea, weight loss, and failure to thrive, most commonly seen in children [8]. In adult populations, “non-classical” CD can present as iron deficiency anemia, constipation, and bloating [9]. These manifestations can be reversed with a gluten-free diet (GFD), although fatigue and other minor GI symptoms may persist [10]. Longer-term complications of undiagnosed CD include cancer, lymphoma, vitamin deficiency, anemia, osteoporosis and infertility [11,12]. A US study on young adults working in the Air Force showed that individuals with undiagnosed CD had a nearly 4-fold increased risk of death during a 45-year follow-up period, compared with those without evidence of CD [13]. Further underlining the importance of making a diagnosis of CD and commencing the patient on a GFD, is the result of a study in Edinburgh showing that those diagnosed with CD in childhood, and treated with a GFD thereafter, had a similar mortality rate to that of the general population [14].

### 1.2. Current Diagnostic Pathway and Its Limitations

The current diagnostic pathway for CD typically involves serological testing for disease-specific antibodies (anti-tissue transglutaminase and anti-endomysial antibodies) followed by the “gold-standard” confirmatory histopathological assessment of endoscopic duodenal biopsies following six weeks of gluten consumption [11]. Heterogeneity in serological testing practices contributes to the diagnostic challenges, with 12 distinct assays being used across 342 UK sites, in addition to marked variability in the upper limit of normal between centers [15]. In most histopathology departments worldwide, pathologists examine the duodenal biopsies histologically, stained simply with hematoxylin and eosin (HE). Hallmark histological features include villous atrophy, crypt hyperplasia, and increased numbers of intraepithelial lymphocytes (IELs) [16], which are the basis of the Marsh–Oberhuber and Corazza–Villanacci classifications (Table 1) [17,18,19]. However, the assessment of these changes, which are part of a continuum, is relatively subjective, even if the Marsh–Oberhuber or Corazza–Villanacci classification is used, leading to diagnostic disagreement between pathologists at least 20–25% of the time [20,21,22,23,24,25,26], contrasting with a 3–6% disagreement rate for biopsies from other body sites [27,28,29]. Furthermore, insufficient gluten consumption prior to biopsy limits the extent to which the classical features of CD are apparent [11], meaning that this patient group is at significant risk of misdiagnosis and may benefit the most from identification of novel CD histological biomarkers. For this reason, in our study, we specifically analyzed biopsies from five known CD patients following a GFD, as these provide an excellent correlate of those gluten-sensitive patients who fail to eat sufficient gluten pre-biopsy, because of the symptoms caused by gluten ingestion. Conversely, the hallmark histological features of CD are not completely exclusive to CD, as they can be seen in Helicobacter or Giardia infection, in viral gastroenteritis and in patients taking certain medications [30]. Consequently, definitive diagnosis relies on the combined interpretation of clinical, serological, and histological findings. There is an unmet need for a more objective and reproducible diagnostic approach for CD.

### 1.3. Immunohistochemistry

Immunohistochemistry (IHC) is a powerful diagnostic technique for the rapid detection of biomarkers with predictive, diagnostic, and prognostic significance [31]. Automated immunostaining platforms are present in most diagnostic histopathology departments worldwide. As a key component in diagnosing CD is the enumeration of IELs, with a diagnostic criterion of >25 IELs per 100 enterocytes, IHC approaches that identify IELs have been reported to improve histological sensitivity for CD compared with HE staining alone [32,33,34].

### 1.4. Candidate Diagnostic Biomarkers Can Be Derived from an Understanding of CD Pathogenesis

Previous studies have demonstrated an increase in the numbers of duodenal gamma delta (γδ) T cells among the IELs in CD, which persists even on a gluten-free diet (GFD) [35,36,37]. These T cells are important for maintaining mucosal integrity and mediating protective immune responses, but they may also be pathogenic, participating in autoimmune responses in CD [26,38]. Alongside these alterations in γδ IEL populations, several associated proteins, including BTNL8, NKp46, TdT, and THEMIS, have been reported to show altered expression in CD, reflecting changes in IEL activation or development, and in epithelial immune signaling.

Butyrophilin-like (BTNL) molecules are increasingly recognized regulators of T-cell immunity and have been identified as activators of γδ T cells in a major histocompatibility complex (MHC)-independent manner [39]. In the intestinal epithelium, BTNL3-BTNL8 complexes interact with local γδ T cell subsets [40]. In CD, reduced BTNL8 expression is accompanied by a loss of BTNL8-responsive Vγ4/Vδ1 T cells. These are replaced by Vδ1 intraepithelial lymphocytes that are unresponsive to BTNL8, a shift that persists on a gluten-free diet [41].

Natural cytotoxicity receptors (NCRs), including NKp46, are immunoglobulin-like receptors classically expressed by natural killer cells [42]. However, previous studies have also identified NCR expression on subsets of γδ T cells, where they contribute to rapid immune responses independent of MHC-restricted antigen presentation [43]. NKp46 has been implicated as a key activating receptor in CD [44,45]. In CD, depletion of NKp46-expressing-Vδ1+ γδ T cells, alongside expansion of NKp46-negative IELs, indicates disease-associated remodeling of the IEL compartment [41]. A permanent loss of NKp46 expression was confined to the duodenum, because colonic IELs regained their NKp46 expression following GFD, indicating a site-specific phenomenon [41].

T-cell receptor (TCR) diversity is generated through V(D)J recombination mediated by recombination-activating genes (RAG) 1/2 and terminal deoxynucleotidyl transferase (TdT) [46]. There is some evidence for limited extrathymic T-cell differentiation in the duodenum [47]. Reduced RAG1 and preT alpha-chain mRNA expression have been reported in untreated celiac disease in IELs from children, suggesting an impaired capacity for extrathymic TCR gene rearrangement [48]. This led us to consider TdT as a potential CD biomarker.

Thymocyte-expressed molecule involved in selection (THEMIS) is a lymphoid-specific gene encoding a T-cell protein that plays a critical role in thymocyte development. It is involved in the regulation of positive selection of thymocytes, enabling appropriate recognition of self-antigens [49]. Genome-wide association studies have linked THEMIS with celiac disease. Additionally, increased duodenal biopsy THEMIS mRNA expression has been reported in patients with active CD compared with healthy controls and those on a gluten-free diet [50].

Because these molecules are involved in immunological processes associated with CD pathogenesis, we tested them as candidate biomarkers for improving histological diagnosis.

### 1.5. Aim of the Study

This study aimed to evaluate the diagnostic performance of five candidate biomarkers, detected by immunohistochemistry (BTNL8, NKp46, TdT, THEMIS, TCRδ), in distinguishing duodenal biopsies from CD patients, either on a gluten-containing diet or GFD, from healthy controls.

## 2. Materials and Methods

### 2.1. Biopsy Selection

Histological material from 46 anonymized duodenal biopsies was obtained from the Human Tissue Research Biobank at Cambridge University Hospitals NHS Foundation Trust, with full ethical approval (IRAS 162057; PI: Professor E. Soilleux). These included 21 cases of confirmed CD on a gluten-containing diet, five cases of confirmed CD on a strict gluten-free diet for at least 6 months, and biopsies from 20 healthy controls. All cases had undergone clinical diagnosis by experienced consultant pathologists at Addenbrookes Hospital, Cambridge, according to standard clinically used criteria [17,18,19,51]. Healthy control biopsies had normal histology, according to the reporting pathologist. The following exclusion criteria were used: CD diagnosis in inpatient or outpatient hospital records, history of malabsorption or diarrhea, anemia, lymphocytosis on biopsy and the patient being on GFD.

### 2.2. Immunohistochemistry

All 46 FFPE biopsies underwent single immunostaining on a Leica BOND-III automated staining platform (Leica Biosystems, Newcastle-upon-Tyne, UK) using the Polymer Refine Detection System (DS9800, Leica Biosystems, Newcastle-upon-Tyne, UK). Sections underwent heat-induced epitope retrieval for 20 min at 100 °C in either sodium citrate buffer (BTNL8) or Tris-EDTA buffer (NKp46, TdT, THEMIS) or epitope retrieval for 40 min in Tris-EDTA buffer (TCRδ). Monoclonal rabbit IgG Clone 2187B anti-BTNL8 antibody (R&D Systems, Minneapolis, MN, USA) was applied at 2.5 µg/mL for 15 min. Mouse monoclonal anti-NKp46/NCR1 antibody (195314, Novus Biologicals, Centennial, CO, USA) was applied at 25 µg/mL for 60 min. Mouse monoclonal anti-TdT antibody (NCL-L-TdT-339, Novocastra, Newcastle-upon-Tyne, UK) was applied at 0.3 µg/mL for 15 min. Rabbit polyclonal anti-THEMIS antibody (NBP3-23438, Novus Biologicals, Centennial, CO, USA) was applied at 0.5 µg/mL for 15 min, and the post-primary mouse linker step was omitted. Mouse monoclonal anti-TCRδ antibody (clone H-41, Santa Cruz Biotechnology, Dallas, TX, USA) was applied at 1:50 dilution for 15 min. Signals were visualized with the Refine detection system with DAB enhancer (AR9432, Leica Biosystems), and slides were counterstained with hematoxylin and mounted in CV Ultra Mounting Medium (14070937891, Leica Biosystems, Newcastle-upon-Tyne, UK).

### 2.3. Image Scanning and Evaluation

The stained slides were scanned using a Leica Aperio AT2 digital slide scanner (Leica Microsystems Ltd., Milton Keynes, UK). Whole-slide images were visualized using PathXL Xplore, version 5.3.0 (Philips, Belfast, UK) and independently evaluated by three observers blinded to diagnosis.

### 2.4. Staining Analysis

Each duodenal biopsy was visually assessed for BTNL8 staining of the surface epithelium and crypt epithelium in each patient sample separately. Each biopsy was scored with a numerical score, reflecting the intensity of the staining as defined in Table 2.

IELs positively immunostained for NKp46, THEMIS, TdT, and TCRδ were quantified, counting bilaterally from the villous tips. In cases with severe villous atrophy, IELs were counted from the flat mucosal epithelium. Five regions of each biopsy with the highest number of stained epithelial cells were selected for scoring. A total of 500 enterocytes were counted per patient for NKp46, and the number of positively stained IELs associated with these 500 enterocytes was given as the mean number per 100 enterocytes. THEMIS, TdT, and TCRδ were expressed at a lower frequency, so a total of 1000 enterocytes were counted per patient. Numbers of positively stained IELs were calculated per 100 enterocytes for each biomarker.

### 2.5. Statistical Analysis and Data Visualization

The intraclass correlation coefficient (ICC) (2,1) was used to determine interobserver agreement and reproducibility [52]. This approach was taken to calculate reliability from a single score, with raters considered representative of a larger population of similar raters. Each biopsy was scored by all three observers, and reliability was calculated from a single quantification.

Descriptive analyses (median and interquartile range (IQR)), group comparisons using Mann–Whitney U tests, interobserver ICC, sensitivity analyses with receiver operator (ROC) curves, and data visualization were all performed using Python Software Foundation (Python Language Reference, version 4.3.1; available at http://www.python.org).

## 3. Results

### 3.1. Analysis of Biomarker Immunostaining

**Figure 1:**
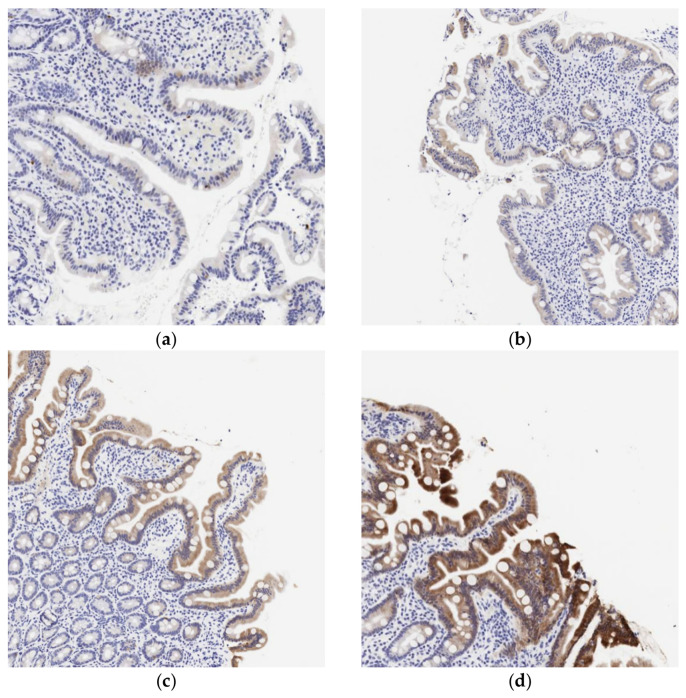
Representative images of BTNL8 staining in duodenal biopsies. (**a**) No staining (0); (**b**) mild staining (1); (**c**) moderate staining (2); (**d**) strong staining (3).

**Figure 2:**
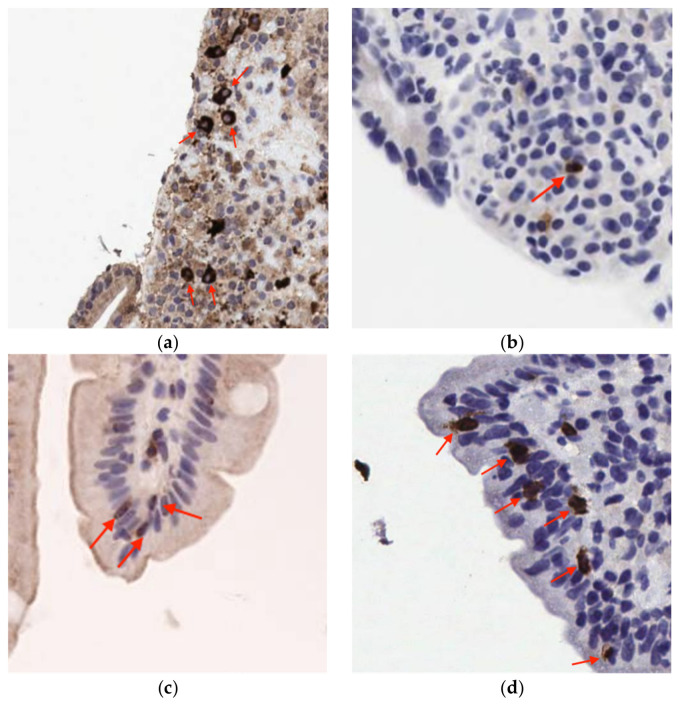
Representative IHC images. (**a**) NKp46; (**b**) TdT; (**c**) THEMIS; (**d**) TCRδ in the duodenum. The red arrows indicate positively stained IELs.

**Figure 3:**
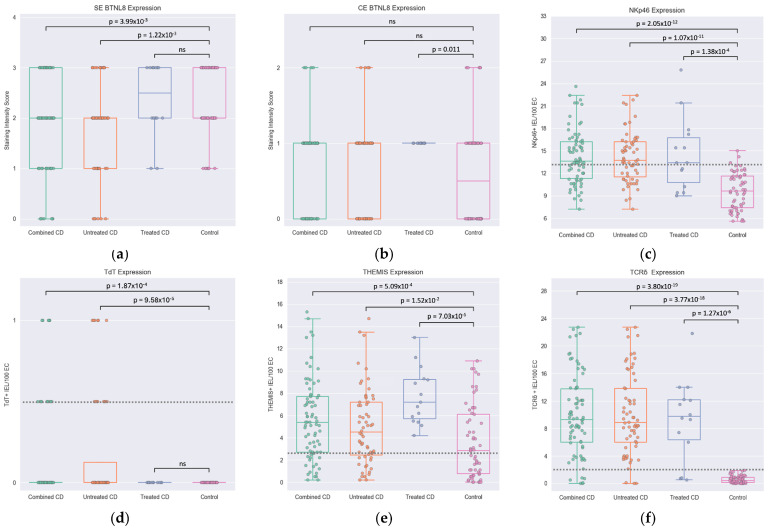
Expression of five immunohistochemical biomarkers across CD subgroups and healthy controls. Boxplots and scatter plots show individual patient values and group distributions, with pairwise comparisons between disease subgroups and controls. Each dot represents the score for a single patient assessed by an individual observer. Gray dotted lines indicate the optimized diagnostic cut-off for each biomarker (summarized in Table 4). (**a**) Surface epithelium BTNL8; (**b**) crypt epithelium BTNL8; (**c**) NKp46; (**d**) TdT; (**e**) THEMIS; (**f**) TCRδ expression. Abbreviations: SE, surface epithelium; CE, crypt epithelium; ns, not significant (at the 0.05 level).

Representative images of BTNL8 immunostaining are shown in Figure 1 and NKp46, THEMIS, TdT, and TCRδ immunostaining in Figure 2. All biopsies were scored by three independent observers, blinded to diagnosis, as described in Section 2.3, giving the results shown in Table 3 and box-and-whisker plots in Figure 3 (raw data included in Appendix A: Table A1, Table A2, Table A3, Table A4, Table A5 and Table A6).

**Table 3:** Immunostaining results for all five biomarkers across the three disease subgroups and controls. BTNL8 staining intensity was enumerated separately on the surface and in crypts on a scaled of 0–3 corresponding to no, mild, moderate and strong staining. IELs positively immunostained for NKp46, THEMIS, TdT, and TCRδ were quantified, counting bilaterally from the villous tips in five regions of each biopsy with the highest number of stained epithelial cells, or in flat mucosal epithelium, if there was severe villous atrophy. IELs associated with 500 enterocytes were counted per patient for NKp46, or with 1000 enterocytes for THEMIS, TdT, and TCRδ. Mean numbers of positively stained IELs are given per 100 enterocytes for each marker. Further information on methods is included in Section 2.3. Raw data corresponding to original cell counts is included in Appendix A: Table A1, Table A2, Table A3, Table A4, Table A5 and Table A6. IQR—interquartile range; ns—no significance (at the 0.05 level).

Diagnostic Group/Observer	Observer 1 Median (IQR)	Observer 2 Median (IQR)	Observer 3 Median (IQR)	Median (IQR) for All Three Observers Combined	*p*-Value Compared with Control
Median surface BTNL8 staining intensity					
Combined CD	2 (1.25–3)	2.5 (2–3)	2 (1–2)	2 (1–3)	0.0039
Untreated CD	2 (1–2)	2 (2–3)	2 (1–2)	2 (1–2)	0.0012
Treated CD	3 (2–3)	3 (3)	2 (2)	2 (2–3)	1 (ns)
Control	2.5 (2–3)	3 (2–3)	2 (1–3)	2 (2–3)	1
Median crypt BTNL8 staining intensity					
Combined CD	1	1 (0.25–1)	1 (0–1)	1 (0–1)	0.16 (ns)
Untreated CD	0	1 (1)	1 (0–1)	1 (0–1)	0.29 (ns)
Treated CD	1	1 (0–1)	1 (1)	1 (1)	0.011
Control	0	1 (0–1)	0 (0–1)	0.5 (0–1)	1
Median TCRδ+ IEL count per 100 enterocytes					
Combined CD	13.9 (8.1–17.8)	8.5 (5.5–10.85)	8.2 (4.5–10.2)	9.4 (6.0–13.9)	3.80 × 10^−19^
Untreated CD	13.9 (7.7–16.9)	8.4 (5.4–11)	8.1 (4–9.6)	8.9 (6.0–13.9)	3.77 × 10^−18^
Treated CD	12.2 (12.1–21.8)	9.5 (6–10)	9.3 (7.4–11.5)	10.0 (6.7–13.1)	1.27 × 10^−6^
Control	0.7 (0.15–1.1)	0.4 (0–0.8)	0.4 (0.2–1)	0.5 (0.1–1.0)	1
Median NKp46+ IEL count per 100 enterocytes					
Combined CD	16.3 (13.9–21.4)	12.6 (10.3–15.4)	13.4 (11.4–15.3)	13.8 (11.7–16.6)	2.05 × 10^−12^
Untreated CD	16.2 (13.6–18.6)	13 (10.6–15.4)	13.4 (11.2–15)	13.8 (11.8–16.4)	1.07 × 10^−11^
Treated CD	21.4 (15.4–25.8)	9.4 (9.4–10.2)	13.4 (13.4–15.4)	13.4 (11.3–17.5)	1.38 × 10^−4^
Control	7.7 (6.95–9.9)	9.9 (8.3–11.6)	10.9 (7.55–11.9)	9.6 (7.4–11.6)	1
Median THEMIS+ IEL count per 100 enterocytes					
Combined CD	6.9 (4.6–8.3)	6.2 (4.3–9.2)	2.75 (1.7–5.3)	5.4 (2.7–7.8)	5.09 × 10^−4^
Untreated CD	5.9 (3.4–7.8)	5.9 (4.1–9.1)	2.5 (1.2–3.6)	4.9 (2.6–7.6)	0.015
Treated CD	7.2 (6.8–9.3)	8.9 (6.2–9.2)	5.4 (5.1–7.9)	7.2 (5.7–9.3)	7.03 × 10^−5^
Control	4.1 (1.0–6.7)	3.4 (0.8–6.6)	1.8 (0.9–3.2)	2.9 (0.8–6.1)	1
Median TdT+ IEL count per 100 enterocytes					
Combined CD	0.0 (0.0)	0.0 (0.0)	0.5 (0.0–1.0)	0.0 (0.0–0.5)	1.87 × 10^−4^
Untreated CD	0.0 (0.0)	0.0 (0.0)	0.5 (0.0–1.0)	0.0 (0.0–0.5)	9.58 × 10^−5^
Treated CD	0.0 (0.0)	0.0 (0.0)	0.5 (0.0–0.5)	0.0 (0.0)	1 (ns)
Control	0.0 (0.0)	0.0 (0.0)	0.0 (0.0)	0.0 (0.0)	1

**Table 4:** Diagnostic performance, thresholds (also shown in Figure 3), and interobserver agreement of five candidate biomarkers ranked by diagnostic performance, calculated as described in Section 2.4.

Marker	ICC (2,1)	Threshold	Sensitivity	Specificity	AUC
TCRδ	0.82	>2.1 TCRδ+/100 EC	0.92	0.95	0.94
NKp46	0.82	>12.8 NKp46+ IEL/100 EC	0.67	0.92	0.86
THEMIS	0.64	>2.2 THEMIS+ IEL/100 EC	0.85	0.45	0.68
TdT	0.22	>0.5 TdT+ IEL/100 EC	0.31	0.90	0.61
BTNL8 CE	0.52	N/A	0.65	0.50	0.56
BTNL8 SE	0.64	N/A	0.00	1.00	0.37

### 3.2. Statistical Evaluation of Immunostained Markers

To determine the potential diagnostic utility of the five biomarkers, including for identifying CD patients on a GFD, results for three disease subgroups were compared with healthy controls. These subgroups were: (1) combined CD, representing both untreated and GFD-treated CD; (2) untreated CD, representing patients on a gluten-containing diet; and (3) GFD-treated CD. We specifically undertook separate analysis of the biopsies from known CD patients following a GFD, as these provide an excellent correlate of those gluten-sensitive patients who fail to eat sufficient gluten pre-biopsy, because of the symptoms caused by gluten ingestion. This is the group of patients that is most likely to receive an inaccurate histopathological diagnosis, because their biopsies may appear histomorphologically normal. They are thus the group most likely to benefit from a novel biomarker.

#### 3.2.1. Statistical Evaluation of BTNL8 Immunostaining Results

Surface and crypt epithelial (SE) BTNL8 staining intensity is summarized in Table 3. Surface BTNL8 intensities were higher in control specimens than in combined CD (*p* = 0.004) or untreated CD (*p* = 0.001), whereas treated CD intensities did not differ significantly from controls. Interobserver agreement for SE BTNL8 scoring was moderate (ICC = 0.64).

Crypt epithelial (CE) BTNL8 expression intensities were low across all groups. Significant differences were observed only between treated CD and controls (*p* = 0.011), with higher intensities in treated CD. Interobserver agreement for CE BTNL8 scoring was lower (ICC = 0.52), indicating modest reproducibility.

#### 3.2.2. Statistical Evaluation of NKp46 Immunostaining Results

Median NKp46-positive IEL counts are summarized in Table 3. All CD subgroups demonstrated higher median counts compared with controls. Pairwise comparisons confirmed significantly elevated NKp46-positive IELs in combined CD (*p* = 2.05 × 10^−12^), untreated CD (*p* = 1.07 × 10^−11^), and treated CD (*p* = 1.38 × 10^−4^) relative to controls. Interobserver agreement was strong (ICC = 0.82), indicating good reproducibility.

#### 3.2.3. Statistical Evaluation of TdT Immunostaining Results

Median TdT-positive IEL counts were low across all groups (Table 3), with slightly more TdT+ IELs being observed in CD. Significant differences compared with controls were observed in combined CD (*p* = 1.87 × 10^−4^) and untreated CD (*p* = 9.58 × 10^−5^), but not in treated CD. Interobserver agreement was poor (ICC = 0.22), indicating limited reproducibility of TdT scoring.

#### 3.2.4. Statistical Evaluation of THEMIS Immunostaining Results

Median THEMIS-positive IEL counts are summarized in Table 3. All CD subgroups demonstrated higher counts compared with controls. Pairwise comparisons showed significantly increased THEMIS-positive IELs in combined CD (*p* = 5.09 × 10^−4^), untreated CD (*p* = 0.015), and treated CD (*p* = 7.03 × 10^−5^) relative to controls. Interobserver agreement was moderate (ICC = 0.64).

#### 3.2.5. Statistical Evaluation of TCRδ Immunostaining Results

Median TCRδ-positive intraepithelial lymphocyte (IEL) counts were markedly elevated in celiac disease compared with controls (Table 3). All CD subgroups demonstrated substantially higher median counts than controls (combined CD (*p* = 3.80 × 10^−19^), untreated CD (*p* = 3.77 × 10^−18^), treated CD (*p* = 1.27 × 10^−6^). Interobserver agreement for TCRδ enumeration was strong (ICC = 0.82), indicating good reproducibility.

### 3.3. Sensitivity Analysis and Diagnostic Performance

**Figure 4:**
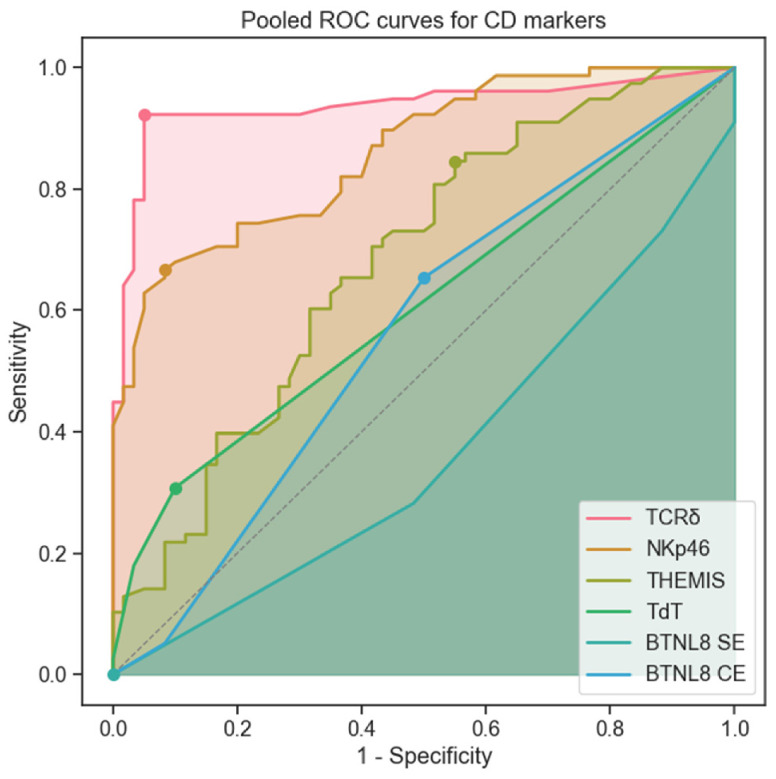
Pooled ROC curves for all six immunohistochemical analyses described in Table 3.

Receiver operating characteristic (ROC) analysis was performed to assess the ability of each biomarker to differentiate CD cases from controls. TCRδ demonstrated the highest diagnostic accuracy, with an area under the ROC curve (AUC) of 0.94, representing the highest discriminatory performance among all markers examined. NKp46 also showed good performance (AUC = 0.86). THEMIS (AUC = 0.68) and TdT (AUC = 0.61) demonstrated limited discriminatory ability. BTNL8 showed limited diagnostic utility, with crypt epithelial staining yielding an AUC of 0.56 and surface epithelial staining an AUC of 0.37. The diagnostic thresholds for each biomarker are shown in Figure 3, and ROC curves for the CD markers are shown in Figure 4.

Diagnostic performance aligned with reproducibility across the three observers. TCRδ and NKp46, which demonstrated the highest AUC values, also showed strong interobserver agreement (ICC = 0.82). In contrast, BTNL8, THEMIS and TdT demonstrated lower discriminatory ability with modest to poor reproducibility despite statistical significance, indicating reduced robustness as markers for CD. Interobserver agreement and diagnostic performance are summarized in Table 4.

## 4. Discussion

### 4.1. Overall Biomarker Utility

This study explored the potential utility in discriminating between celiac and non-celiac biopsies of five different immunohistochemically detected biomarkers (BTNL8, NKp46, TdT, THEMIS, and TCRδ). They were selected given their potential associations with CD pathogenesis. We compared their expression in active CD and GFD-treated CD with that in non-celiac controls. Some CD patients are re-biopsied after 6 months of a GFD. These GFD-treated CD biopsies are an excellent correlate of the initial CD diagnostic biopsies received from patients who have consumed insufficient gluten prior to biopsy, because of the unpleasant symptoms that eating gluten causes them. This particular patient group is the most likely group to have mild or minimal changes of CD in their biopsies, with an attendant risk of the diagnosis of CD being missed. New biomarkers to improve diagnostic accuracy would be of the greatest value in this group. In addition, because some patients may have insufficient gluten intake pre-biopsy, if gluten ingestion causes them severe symptoms, the combined untreated CD and treated CD group may be more representative of current biopsy case mixes.

### 4.2. Utility of TCRδ Immunostaining

Numbers of TCRδ+ IEL were significantly higher in GFD-treated and active CD biopsies compared with healthy controls, consistent with the known expansion of γδ T cells in CD, that persist even after a GFD [35,36,37]. Results are also consistent with a previous immunohistochemical study using a now discontinued anti-TCRγ clone, which would have identified the same T-cell populations as the anti-TCRδ antibody used here [53]. The ICC score of 0.82 indicated good interobserver agreement and TCRδ was the biomarker achieving the highest AUC (0.94). The robust performance of TCRδ in this study suggests its utility as a reproducible biomarker for identifying CD, even in biopsies from patients who have consumed insufficient gluten prior to biopsy. Indeed, its ability to distinguish GFD-treated CD biopsies from healthy controls means that TCRδ immunostaining might eliminate the need for gluten consumption pre-biopsy, if additional larger studies GFD-treated CD biopsies corroborate our findings. At the very least, IHC for this biomarker could substantially improve agreement between pathologists, by providing a quantitative threshold for IEL assessment, thereby complementing the existing gold-standard diagnostic test. A larger multi-center study will be needed to confirm the figure of >2.1 TCRδ+ IEL per 100 enterocytes, not least because factors such as section thickness and staining intensity might confound the assessment of numbers of IEL per 100 enterocytes.

### 4.3. Utility of NKp46 Immunostaining

NKp46 expression was increased in CD biopsies, contradicting a previous report of NKp46 depletion at sites of mucosal damage in CD [41]. This discrepancy may reflect methodological differences, as Mayassi et al. [41] used flow cytometry, whereas this study used IHC analysis of tissue sections. Given that NKp46 is expressed by a subset of γδ T cells, the expansion of γδ T cells in CD, which our TCRδ immunostaining in this study confirms, could result in increased NKp46 expression, even if the proportion of NKp46-positive T cells is actually reduced. Interpretation of NKp46 was challenging, due to high levels of background staining, and scoring was therefore restricted to strongly stained IELs to minimize subjectivity. Further work is required to determine whether the observed increase in NKp46 expression in CD reflects a true biological change or a methodological limitation.

### 4.4. Utility of BTNL8 Immunostaining

BTNL8 staining in both the surface and crypt epithelium showed limited diagnostic utility, with poor to moderate interobserver agreement and modest AUC values. There were significantly lower levels of expression by the duodenal surface epithelium in untreated CD than GFD-treated CD and controls, consistent with previous work by Mayassi and colleagues [41], although somewhat less clearcut than suggested by this study. BTNL8 expression on the crypt epithelium showed little difference between active CD and controls, although levels in the crypts of treated CD patient biopsies were slightly higher than those of controls (*p* = 0.011).

### 4.5. Utility of THEMIS Immunostaining

Although THEMIS showed statistically significant differences between CD and control biopsies, independent of dietary gluten intake, the changes in expression changes were relatively subtle, with moderate discriminatory performance (AUC = 0.68) and limited interobserver agreement (ICC = 0.64), likely indicating limited reproducibility. One previous RT-PCR-based study using RNA from whole duodenal biopsies reported increased THEMIS expression in CD compared with controls [50], in agreement with our data. However, the relatively subtle differences seen in IHC and limited interobserver agreement are likely to preclude THEMIS being a useful diagnostic marker for CD.

### 4.6. Utility of TdT Immunostaining

TdT quantification was difficult due to the extremely small numbers of positive cells resulting in very limited interobserver agreement (ICC = 0.22) and poor discriminatory performance between CD and controls (AUC = 0.61). Increased expression of TdT-positive IELs was observed in untreated coeliac disease, in contrast to previous reports demonstrating reduced RAG1 mRNA expression in patients with coeliac disease compared to controls [48]. However, this may have been confounded by the expansion of lymphocytes present in CD compared to controls, which could influence overall cell counts.

The two previous studies considering extrathymic T-cell differentiation in the duodenum focused on the expression of mRNA encoding RAG1, RAG2 and the preT alpha-chain [47,48]. When considering the machinery of T-cell differentiation, we elected to detect TdT because of the very robust antibody available against this target. To our knowledge, there are no publications describing TdT mRNA expression in the duodenum, precluding corroboration of our findings. Notably, all the biopsies in our study were from adults, while the prior RT-PCR study reporting decreased RAG1 and preT alpha-chain expression in untreated CD were performed using IELs from children [48] and there may be age-related changes in the levels of extrathymic T-cell differentiation, with the process being more prominent in children. It may therefore be helpful to evaluate TdT expression in a pediatric CD and control biopsy cohort, expressed as a percentage of total lymphocytes, to account for both age-related differences and the expansion of intraepithelial lymphocytes observed in CD.

### 4.7. Study Limitations and Future Work

Our study suffers from several limitations. The total numbers of biopsies were relatively small, but the GFD-treated group contained biopsies from only five patients. Furthermore, all the biopsies we used were from adults. Future work should prioritize validation of TCRδ IHC in larger, multicenter cohorts across all patient ages, including children, particularly in GFD-treated patients and in individuals with gastrointestinal conditions that may mimic celiac disease. To maximize diagnostic reproducibility and make best use of pathologists’ time and expertise, artificial intelligence (AI) could be applied to histopathological whole-slide images immunostained for TCRδ, alongside a serial HE image, using an enumeration approach, similar to that in our recent work and that of others [54,55].

## 5. Conclusions

This study evaluated the potential diagnostic utility of five candidate biomarkers in celiac disease to address the challenges associated with classical histological diagnosis, particularly in biopsies with relatively equivocal histological changes due to insufficient gluten intake or co-existing GI conditions. Overall, TCRδ emerged as the most promising marker in this study, making it a potential adjunct to the current histological gold-standard biopsy assessment, particularly in cases with borderline changes where diagnostic confidence is low. This would help determine its utility in cases with insufficient gluten exposure and histologically ambiguous biopsies, potentially reducing interobserver variability and improving diagnostic accuracy. Validation in larger, multi-center cohorts will be required to establish the robustness of these findings, particularly in biopsies from GFD-treated patients and children. By introducing more objective, biomarker-supported thresholds for intraepithelial lymphocyte assessment, this approach has the potential to reduce inter-pathologist variability, minimize diagnostic ambiguity, and ultimately improve the clinical pathway for patients with histologically subtle celiac disease.

## Figures and Tables

**Table 1:** The two most commonly used histological classification systems for CD [17,18,19].

Modified Marsh–Oberhuber Classification				Corazza–Villanacci Classification	Comments
Type	IELs (/100 Enterocytes)	Crypt Hyperplasia	Villous Atrophy	Grade	Clinical Notes
**Type 0**	<25/100	No	No	Normal	Normal mucosa; celiac disease highly unlikely
**Type 1**	≥25/100	No	No	Grade A (non-atrophic)	Increased IELs only; seen on GFD, dermatitis herpetiformis, family members of CD patients; not specific (infections, NSAID use, *Helicobacter pylori*)
**Type 2**	≥25/100	Yes	No	Grade A (non-atrophic)	Increased IELs + crypt hyperplasia; rare; may be seen in dermatitis herpetiformis. Corazza–Villanacci merges Types 1 and 2 as both are non-atrophic
**Type 3a**	≥25/100	Yes	Mild (partial)	Grade B1 (atrophic; V:C < 3:1)	Mild villous atrophy; villus:crypt ratio < 3:1; symptomatic CD spectrum
**Type 3b**	≥25/100	Yes	Marked (subtotal)	Grade B1 (atrophic; V:C < 3:1)	Subtotal villous atrophy; villus:crypt ratio < 3:1; symptomatic CD spectrum
**Type 3c**	≥25/100	Yes	Total (flat mucosa)	Grade B2 (atrophic; villi absent)	Total villous atrophy; flat mucosa; villi no longer identifiable; typical untreated symptomatic CD
**Type 4**	Variable	No	Total (flat mucosa)	—	Hypoplastic atrophy (flat mucosa without crypt hyperplasia); extremely rare; associated with refractory CD or T-cell lymphoma; not included in Corazza–Villanacci

**Table 2:** Scoring system for BTNL8 staining intensity.

Score	Definition
0	No staining
1	Mild staining
2	Moderate staining
3	Strong staining

## Data Availability

The original contributions presented in this study are included in the article. Further inquiries can be directed to the corresponding author.

## Abbreviations

The following abbreviations are used in this manuscript:

AI	Artificial intelligence
AUC	Area under the ROC curve
BTNL	Butyrophilin-like
CD	Celiac disease
CE	Crypt epithelium
GFD	Gluten-free diet
HE	Hematoxylin and eosin
ICC	Intraclass coefficient
IEL	Intraepithelial lymphocytes
IHC	Immunohistochemistry
IQR	Interquartile range
NCR	Natural cytotoxicity receptors
RAG	Recombination-activating genes
ROC	Receiver operating characteristic
SE	Surface epithelium
TCR	T-cell receptor
TdT	Terminal deoxynucleotidyl transferase
THEMIS	Thymocyte-expressed molecule involved in selection
tTG	Tissue transglutaminase
γδ	Gamma-delta

## Appendix A

**Table A1:** BTNL8 surface epithelium scoring data for all 46 patients. Observers 1–3 independently assessed each biopsy, blinded to diagnosis.

Patient	CD Group	Observer 1	Observer 2	Observer 3
1	CD untreated	2	2	1
2	CD untreated	2	2	2
3	CD untreated	1	2	1
4	CD untreated	2	3	2
5	CD untreated	1	1	1
6	CD untreated	2	3	2
7	CD untreated	3	2	2
8	CD untreated	2	2	2
9	CD untreated	3	3	3
10	CD untreated	2	2	2
11	CD untreated	0	0	0
12	CD untreated	3	3	2
13	CD untreated	2	3	2
14	CD untreated	2	3	1
15	CD untreated	2	3	1
16	CD untreated	1	2	1
17	CD untreated	1	2	1
18	CD untreated	2	2	2
19	CD untreated	3	3	2
20	CD untreated	3	3	2
21	CD untreated	0	0	0
22	CD GFD treated	3	3	2
23	CD GFD treated	3	3	2
24	CD GFD treated	0	1	1
25	CD GFD treated	2	3	2
26	CD GFD treated	3	3	2
27	Controls	3	3	3
28	Controls	3	3	2
29	Controls	2	2	1
30	Controls	2	3	3
31	Controls	2	2	1
32	Controls	3	3	3
33	Controls	3	3	2
34	Controls	3	3	3
35	Controls	2	2	1
36	Controls	2	2	1
37	Controls	2	2	2
38	Controls	2	3	2
39	Controls	2	2	1
40	Controls	3	3	2
41	Controls	3	3	3
42	Controls	3	3	2
43	Controls	3	3	2
44	Controls	2	3	1
45	Controls	3	3	3
46	Controls	2	2	1

**Table A2:** BTNL8 crypt epithelium scoring data for all 46 patients. Observers 1–3 independently assessed each biopsy, blinded to diagnosis.

Patient	CD Group	Observer 1	Observer 2	Observer 3
1	CD untreated	1	1	0
2	CD untreated	0	0	0
3	CD untreated	0	0	0
4	CD untreated	1	1	1
5	CD untreated	1	1	1
6	CD untreated	2	2	1
7	CD untreated	0	1	1
8	CD untreated	0	0	1
9	CD untreated	1	1	1
10	CD untreated	0	1	1
11	CD untreated	0	1	0
12	CD untreated	1	1	1
13	CD untreated	0	1	0
14	CD untreated	0	1	0
15	CD untreated	1	1	1
16	CD untreated	0	1	0
17	CD untreated	1	2	1
18	CD untreated	1	1	1
19	CD untreated	0	0	1
20	CD untreated	1	2	1
21	CD untreated	0	0	0
22	CD GFD treated	1	0	1
23	CD GFD treated	1	0	1
24	CD GFD treated	1	1	1
25	CD GFD treated	0	1	1
26	CD GFD treated	1	1	1
27	Controls	1	1	1
28	Controls	1	1	1
29	Controls	0	1	0
30	Controls	2	2	1
31	Controls	0	0	0
32	Controls	1	1	0
33	Controls	0	1	0
34	Controls	2	2	2
35	Controls	0	0	0
36	Controls	1	0	1
37	Controls	0	0	0
38	Controls	0	0	0
39	Controls	1	1	1
40	Controls	0	0	1
41	Controls	0	1	0
42	Controls	0	1	1
43	Controls	1	1	0
44	Controls	0	0	0
45	Controls	1	1	1
46	Controls	0	0	0

**Table A3:** NKp46 scoring data for all 46 patients. Observers 1–3 independently assessed each biopsy, blinded to diagnosis.

Patient	CD Group	Observer 1	Observer 2	Observer 3
1	CD untreated	21.8	16.4	19.6
2	CD untreated	11.6	11	11.2
3	CD untreated	12	12.4	9.8
4	CD untreated	13.6	13.2	12
5	CD untreated	16.8	15.4	15
6	CD untreated	15.6	13	21.2
7	CD untreated	15.2	12.8	17.2
8	CD untreated	14.8	15.4	15
9	CD untreated	23.6	15.8	18.8
10	CD untreated	16.2	13.4	13.2
11	CD untreated	15	10.6	13.4
12	CD untreated	13.4	10.6	14
13	CD untreated	18.6	13.8	11
14	CD untreated	22.4	9.8	13.8
15	CD untreated	36.6	35.6	16.8
16	CD untreated	16.4	12	8.4
17	CD untreated	18.6	16.2	14.8
18	CD untreated	16.6	10.6	7.2
19	CD untreated	10.6	8.6	11.2
20	CD untreated	13.6	10.2	11.2
21	CD untreated	21.4	16	13
22	CD GFD treated	35.6	9.4	15.4
23	CD GFD treated	21.4	9.4	12.4
24	CD GFD treated	12.6	9	13.4
25	CD GFD treated	25.8	17.8	13.4
26	CD GFD treated	15.4	10.2	17.2
27	Controls	6.8	5.8	13
28	Controls	9.4	6.6	11.6
29	Controls	14.2	12.2	12.2
30	Controls	6.2	9.2	10.6
31	Controls	9.2	12.4	15
32	Controls	10.8	10.8	12.4
33	Controls	9.6	11.4	11.4
34	Controls	7.4	13.4	11.2
35	Controls	7	11.2	6.6
36	Controls	12.4	8	10.4
37	Controls	7	7.6	13
38	Controls	5.6	8.4	9.8
39	Controls	7	12.6	8.6
40	Controls	7.8	11.6	11.6
41	Controls	8.2	9.6	7.6
42	Controls	12.4	7.6	5.6
43	Controls	11.8	10	11.8
44	Controls	5.6	9.8	7.4
45	Controls	6.4	11.6	7
46	Controls	7.6	9.4	6.6

**Table A4:** TdT scoring data for all 46 patients. Observers 1–3 independently assessed each biopsy, blinded to diagnosis.

Patient	CD Group	Observer 1	Observer 2	Observer 3
1	CD untreated	0	0	0
2	CD untreated	1	1	0.5
3	CD untreated	0	0	0.5
4	CD untreated	0	0	0.5
5	CD untreated	0	0	1.5
6	CD untreated	1.5	1.5	1
7	CD untreated	0	0	0.5
8	CD untreated	0	0	0
9	CD untreated	0	0	2.5
10	CD untreated	0	0	1.5
11	CD untreated	0	0	0
12	CD untreated	0	0	0
13	CD untreated	1	1	1.5
14	CD untreated	0	0	0.5
15	CD untreated	0	0	0
16	CD untreated	0	0	0
17	CD untreated	0	0	0.5
18	CD untreated	0	0	1
19	CD untreated	0	0	0.5
20	CD untreated	0	0	1.5
21	CD untreated	0	0	0.5
22	CD GFD treated	0	0	0
23	CD GFD treated	0	0	0.5
24	CD GFD treated	0	0	2.5
25	CD GFD treated	0	0	0
26	CD GFD treated	0	0	0.5
27	Controls	0	0	0
28	Controls	0	0	0
29	Controls	0	0	0
30	Controls	0	0	0
31	Controls	0	0	0
32	Controls	0	0	0.5
33	Controls	0	0	0
34	Controls	0	0	0
35	Controls	0	0	0
36	Controls	0	0	0
37	Controls	0	0	0
38	Controls	0	0	0
39	Controls	0	0	1.5
40	Controls	0.5	0.5	0
41	Controls	0	0	0
42	Controls	0	0	0
43	Controls	0	0	0
44	Controls	0	0	1
45	Controls	0	0	0
46	Controls	0	0	0.5

**Table A5:** THEMIS scoring data for all 46 patients. Observers 1–3 independently assessed each biopsy, blinded to diagnosis.

Patient	CD Group	Observer 1	Observer 2	Observer 3
1	CD untreated	2.7	3.1	1.2
2	CD untreated	0.7	1.4	0.2
3	CD untreated	2.3	4.3	0.2
4	CD untreated	3.4	6.1	0.9
5	CD untreated	7	13.5	4.9
6	CD untreated	20	26.5	10.7
7	CD untreated	1.5	2.7	2.5
8	CD untreated	14.7	15.3	7.8
9	CD untreated	7.8	10.2	3.6
10	CD untreated	9.3	6.9	2
11	CD untreated	5.1	4.4	2.8
12	CD untreated	5.4	7.6	0.8
13	CD untreated	5.9	5.1	2.6
14	CD untreated	7.7	5.9	3.2
15	CD untreated	8.4	5.4	2.2
16	CD untreated	8.9	9.1	1.6
17	CD untreated	4.6	3.5	5.8
18	CD untreated	0.5	4.1	0.5
19	CD untreated	5.2	3.7	2.2
20	CD untreated	7.2	13.2	2.7
21	CD untreated	6.9	7.5	7.2
22	CD GFD treated	6.8	9.2	4.2
23	CD GFD treated	5.9	5.5	5.1
24	CD GFD treated	9.3	6.2	7.9
25	CD GFD treated	7.2	8.9	5.4
26	CD GFD treated	11.2	13	10.4
27	Controls	10.2	9.7	3
28	Controls	8.6	8.6	2.8
29	Controls	4.2	5.3	0.7
30	Controls	2.3	1.1	0.3
31	Controls	6.7	6.7	2.9
32	Controls	5.4	3.9	1.9
33	Controls	6.2	6.1	3.9
34	Controls	9.9	10.2	3.1
35	Controls	4.5	4.5	1.7
36	Controls	4	2.9	5.6
37	Controls	6.7	6.5	8.3
38	Controls	0.5	0.6	1.1
39	Controls	8.1	7.1	10.9
40	Controls	1.6	1.8	3.3
41	Controls	0	0	0
42	Controls	1.1	0.8	0.9
43	Controls	0.6	0.5	1.7
44	Controls	0.2	0	0.1
45	Controls	0	0	0.2
46	Controls	2.4	2.1	1.1

**Table A6:** TCRδ scoring data for all 46 patients. Observers 1–3 independently assessed each biopsy, blinded to diagnosis.

Patient	CD Group	Observer 1	Observer 2	Observer 3
1	CD untreated	18.2	11	17.8
2	CD untreated	6.7	5.2	3.4
3	CD untreated	12	9.2	6.1
4	CD untreated	13.8	10.1	7.7
5	CD untreated	3.7	4	3.5
6	CD untreated	9.4	5.9	6.5
7	CD untreated	11.6	8.4	8.1
8	CD untreated	22.7	21.5	12.1
9	CD untreated	18.1	12.1	18.9
10	CD untreated	7.7	3.4	3.6
11	CD untreated	22.4	21.3	17.4
12	CD untreated	7.3	5.4	4
13	CD untreated	18.8	8.2	9.6
14	CD untreated	0	0	0.1
15	CD untreated	15	8.4	10.4
16	CD untreated	16	11.6	9.6
17	CD untreated	13.9	12.4	8.2
18	CD untreated	16.7	8.5	8.9
19	CD untreated	16.6	10.4	6.9
20	CD untreated	3.8	3	2.1
21	CD untreated	16.9	8.3	8.8
22	CD GFD treated	26.1	10	14
23	CD GFD treated	12.2	9.5	7.4
24	CD GFD treated	21.8	14	11.5
25	CD GFD treated	12.1	6	9.3
26	CD GFD treated	0.7	0.5	0.8
27	Controls	0	0	0
28	Controls	1.1	1.6	1.3
29	Controls	0	0	0.2
30	Controls	0.3	0	0.1
31	Controls	0	0.1	0.2
32	Controls	0.2	0	0.1
33	Controls	1.1	1.1	1.8
34	Controls	7.7	5.3	9.8
35	Controls	0.8	0.9	0.5
36	Controls	0.7	0.7	0.7
37	Controls	1.9	0.7	0.8
38	Controls	1.2	0.8	1
39	Controls	0.6	0	0.1
40	Controls	1.7	1.3	1.4
41	Controls	0.5	0.3	0.3
42	Controls	0.9	0.4	0.6
43	Controls	0.7	0.7	1
44	Controls	0	0	0.1
45	Controls	0	0.1	0.2
46	Controls	0.3	0.2	0.3
